# Stem Cell Therapy for Erectile Dysfunction: A Step towards a Future Treatment

**DOI:** 10.3390/life13020502

**Published:** 2023-02-11

**Authors:** Xabier Pérez-Aizpurua, María Garranzo-Ibarrola, Carlos Simón-Rodríguez, Juan Vicente García-Cardoso, César Chávez-Roa, Leticia López-Martín, Jaime Jorge Tufet i Jaumot, Josué Alonso-Román, Jesús Maqueda-Arellano, Blanca Gómez-Jordana, Joaquín Ruiz de Castroviejo-Blanco, Felipe Osorio-Ospina, Carmen González-Enguita, Mariano García-Arranz

**Affiliations:** 1 Urology Department, Hospital Universitario Fundación Jiménez Díaz, 28040 Madrid, Spain; 2 Urology Department, Hospital Universitario Virgen de la Macarena, 41009 Sevilla, Spain; 3 Instituto de Investigación Sanitaria (IIS-FJD), Hospital Universitario Fundación Jiménez Díaz, 28040 Madrid, Spain

**Keywords:** erectile dysfunction (ED), stem cell therapy, mesenchymal stem cells (MSC), bone marrow-derived stem cells (BMSC), adipose-derived stem cells, intracavernous injection (ICI)

## Abstract

*Background*: The improvement of absent or partial response in the medical treatment of erectile dysfunction (ED) has led to the development of minimally invasive new treatment modalities in the field of regenerative medicine. *Methods*: A literature review on stem cell therapy for the treatment of ED was performed. We searched for the terms “erectile dysfunction” and “stem cell therapy” in PubMed and Clinicaltrials.gov. Literature searching was conducted in English and included articles from 2010 to 2022. *Results*: New treatment modalities for ED involving stem cell therapy are not only conceived with a curative intent but also aim to avoid unnecessary adverse effects. Several sources of stem cells have been described, each with unique characteristics and potential applications, and different delivery methods have been explored. A limited number of interventional studies over the past recent years have provided evidence of a safety profile in their use and promising results for the treatment of ED, although there are not enough studies to generate an appropriate protocol, dose or cell lineage, or to determine a mechanism of action. *Conclusions*: Stem cell therapy is a novel treatment for ED with potential future applications. However, most urological societies agree that further research is required to conclusively prove its potential benefit.

## 1. Introduction

**Figure 1:**
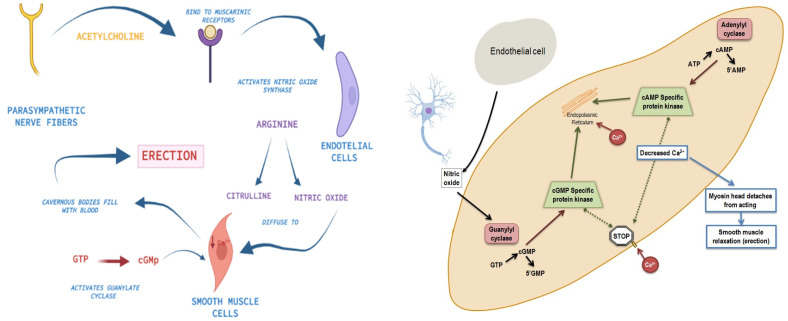
Mechanism of erection and pathophysiology of erectile dysfunction.

Erectile dysfunction (ED), defined as the inability to initiate or maintain an erection satisfactory for sexual intercourse, is the most prevalent male sexual disorder [1]. An estimated prevalence of 50% among men between the ages of 40 and 70 is generally accepted, with almost 15% of patients reporting complete ED and the rest experiencing any degree of ED [2]. The increase in the modifiable risk factors for ED, such as cardiovascular disease, atherosclerosis, metabolic syndrome, diabetes mellitus or prostate cancer, has led to an increase in its prevalence in recent years [3]. Excluding ED of psychogenic origin, organic DE is the result of physical damage to vascular, endocrine, neurologic and anatomical structures, all of which comprise an intricate network of structural pathways which require the integration of signals from nerves, cavernous muscle and endothelium to produce a penile erection [2]. A diagram of biochemical signaling at a cellular level during erection and the pathophysiology of ED is depicted in Figure 1.

The mechanism by which MSC exert their action is unknown. Stem cells are believed to be capable of differentiating into various cell types, including endothelial cells, smooth muscle cells, Schwann cells and neuron cells. The intracavernous injection of MSC might replace the endothelial and/or cavernous smooth muscle cell damage. Other proposed theories include the paracrine effect produced by MSC after injection, rather than cellular differentiation. There are studies studying the use of MSC and the regulation of certain key mediators in the mechanism of erection, including intracellular nitric oxide and calcium concentrations. Regulation of both nitric oxide and calcium intracellularly via the action of various transmembrane transport ionic channels is believed to be the therapeutic target of stem cell therapy in ED; however, the exact mechanism is still unknown [4,5,6,7].

Despite the prevalence of ED, together with its clinical importance and the associated functional and psychological burden in patients affected by this condition, recent data suggest that its real prevalence is significantly underrated. This is particularly notable in the younger population, mainly due to understatement of their condition, with a presumed proportion of up to 10–30% [8]. Moreover, also alarming is the low proportion of patients diagnosed with ED and, subsequently, those actively treated, with about half of these patients receiving medical treatment for their condition. Medical treatment for ED comprises a broad spectrum of therapies with phosphodiesterase 5 inhibitors (PDEi) as the most commonly used drugs due to overall efficacy and few contraindications [9].

ED refractory in medical therapy is increasingly more common nowadays due to the advent of the modifiable risk factors previously stated. More precisely, diabetic patients and post-prostatectomy patients represent a high proportion of these PDEi non-responders [10]. Nerve-sparing prostatectomy, despite new techniques such as robotic assisted surgery (RARP) and noble preserving techniques, can lead to ED in up to 90% of patients undergoing surgery for prostate cancer, as a result of direct neural damage or indirect neuropraxia [11]. Peyronie’s Disease, a chronic condition characterized by the formation of fibrous plaques in the tunica albuginea, leading to penile deformation associated with veno-occlusive ED, also represents a proportion of PDEi non-responders [12]. These stated conditions lead to irreparable damage in the main physical mechanism responsible for erection; thus, it seems credible to assume that PDEi, which exert their action via enhancing intracellular signaling, are not effective.

During the last decade, new therapeutical modalities not only aiming at symptomatic treatment but also with a curative intent have been approached [13,14]. Among these new treatment modalities, shockwave therapy, platelet-rich plasma injections and stem cell therapy (SCT) are included [15,16]. Following advances on stem cell research, it has been postulated that stem cells could differentiate into endothelial, neural and smooth muscle cells in order to replace damage to the tissue of penile structures associated with ED [17,18,19]. The in vitro differentiation into these cellular lines has shown promising results in a preclinical setting in various animal models; however, the main source of their therapeutic effect seems to be related to the paracrine effect they enhance in surrounding tissues [20].

Different sources of stem cells have been used over the years. Mesenchymal stem cells (MSC) are a pluripotent adult stem cell population capable of both self-renewal and differentiation into multiple cell lines and represents the most widely studied and used cellular group for the treatment of ED [21,22]. It is the cellular line with the least ethical considerations derived from its use and with the least oncogenic potential due to its genetic stability compared with other cellular sources [23]. MSC can be obtained from a variety of cellular populations present in the organism. Bone marrow-derived stem cells (BMSC), adipose-derived stem cells (ADSC), amniotic fluid stem cells (AFSC), placental stem cells (PSC) and urine-derived stem cells have all been used in animal or human research in the context of ED management.

## 2. Materials and Methods

A literature search was performed aiming to review the most relevant available literature on the current status of mesenchymal stem cell therapy applied to the field of ED management. Systematic reviews, clinical trials and human studies were prioritized. In the case of basic research review, mainly on a preclinical setting, we focused on studies with in vivo experimentation in animal models.

**Figure 2:**
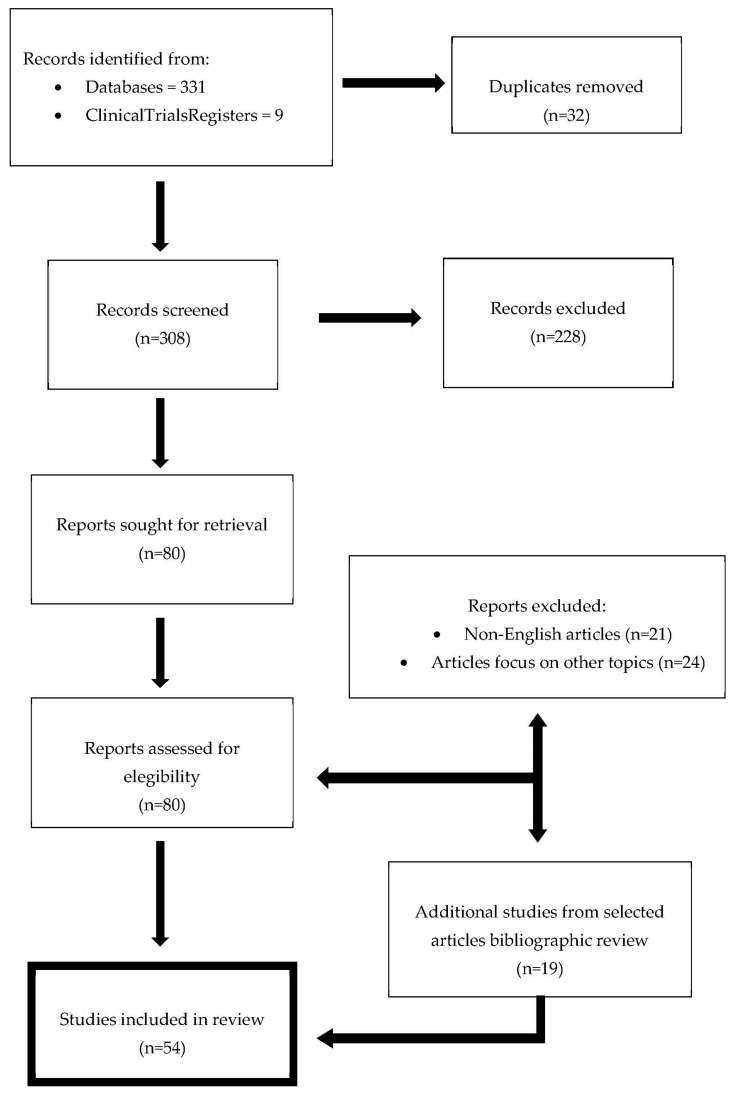
Search strategy flow diagram.

A search strategy (Figure 2) was initially defined following the PRISMA statement recommendations in the Medline database. Simultaneously, the ongoing, future and completed clinical trials registered in ClinicalTrials.gov were identified. The following relevant keywords (erectile disfunction) and (stem cell therapy) were used to conduct a search in PubMed.

A total of 370 results were retrieved. Two reviewers (X.P and M.G) scanned for duplicates and selected the desired studies for further research. Only manuscripts in English were considered. After initial individual review, the most relevant studies prioritizing high-level evidence were selected for final review. Additional relevant studies were included following the previously selected works’ bibliographic review. A final review of 54 studies was performed.

## 3. Results

### 3.1. Stem Cell Therapy for ED in the Preclinical Setting

In the year 2004, Bochinski et al. first described the potential therapeutical benefit of neural crest-derived stem cells in a rat model with ED of neurogenic origin [24]. Since then, a large number of studies in a preclinical setting have aimed to prove the efficacy of stem cell therapy for the treatment of ED secondary to different etiologies: aging, diabetes mellitus, hypertriglyceridemia, cavernous nerve injury and others. There are four published meta-analyses which cover the available evidence in a preclinical setting to this day [25,26,27,28].

In the first work, the final analysis of the 12 included studies allowed Shan et al. to prove the efficacy of stem cell therapy in rat models with cavernous nerve injury (CNI). Further subgroup analysis revealed that observed effects were not associated with the models of CNI simulated and, rather, seemed to be associated with the duration of follow-up, stem cell source and cellular delivery method. Additionally, uncultured stem cells were poorly effective compared with cultured stem cells [26].

In 2017, Hou et al. further reviewed the available evidence on the use of adipose-derived stem cells (ADSC) in ED rat models other than CNI, including other etiologies such as diabetes mellitus, smoking, radiotherapy and tunica albuginea injury. Meta-analysis results revealed that ADSC use can regenerated damaged erectile tissues and aid in the recovery of erectile function. Subsequent findings include a series of concomitant substances secreted by these cells, therefore exerting a kind of paracrine effect which in subgroup analysis significantly improved erectile function in comparison with ADSC alone. The identified substances include neurotrophic factors such as brain derived neurotrophic factor, vascular endothelial growth factor (VEGF) and hepatocyte growth factor. Regarding diabetic rat models, the infusion of a large of ADSC (>1 × 10^6^), ADSC infusion combined with insulin therapy and the previous hypoxic conditioning of ADSC revealed better outcomes [27]. In line with previous research in the field, Park et al. in a meta-analysis published in 2019 analyzed 19 studies with the aim of evaluating the effects of ADSC in rat models with CNI-induced ED. The main findings revealed that ADSC therapy improved erectile function based on penile hemodynamic parameters, intracavernous pressure and mean arterial penile pressure proportion (ICP/MAP), and that ADSC were able to regenerate damaged tissues following changes in measured levels of nitric oxide synthase (NOS), cyclic guanosine monophosphate (cGMP) and smooth muscle-to-collagen ratio [28].

Similar results are described in a recently published network meta-analysis by Wani et al. aimed at evaluating the role of stem cell therapy in erectile dysfunction secondary to cavernous nerve injury [25]. Results including 29 animal studies and three human trials (post-RP patients) revealed a significant increase in histological and molecular parameters of response, including nNOS, smooth muscle content and anti-apoptotic activity in rats, and an improvement in IEEF and EHS in human studies.

Studies in a preclinical setting have not been able to elucidate the exact specific mechanism by which stem cell therapy exerts its therapeutical effect. However, a paracrine effect derived from their use with the secretion of growth factors with chemotactic, anti-inflammatory, regenerative, angiogenic and antiapoptotic properties has been proposed which favors the regeneration of damaged tissues [29,30,31,32,33]. Preclinical research studies have demonstrated an improvement in the vascular function of cavernous endothelium and the suppression of cavernous fibrosis with an increase in the proportion of smooth muscle, derived from stem cell use [34,35]. Furthermore, the use of ADSC also leads to a neurological improvement at the level of cavernous bodies, which is crucial for the maintenance of erectile function [36,37] and a reduction in apoptotic and inflammatory response levels related to increasing ED risk factors such as diabetes mellitus or obesity [34,38].

During 2022, new preclinical studies on stem cell therapy for erectile dysfunction have been published, and new findings on treatment response markers have been made in several animal studies.

Feng et al. observed reduced oxidative stress levels and tissue iron content in diabetic rats treated with UCSC with a concomitant overexpression of ferroptosis inhibitory genes SLC7A11 and GPX4. They therefore concluded that stem cell therapy could exert part of its therapeutical effect by attenuation of diabetic-induced ferroptisis [39]. Similarly, in another diabetic rat model study treated with ADSC designed by Luo et al., a suppression of NLRP3-mediated pyroptosis was observed, suggesting a possible pathogenic mechanism of ED in DM [40]. Other treatment response markers recently proposed include TGF-β, α-SMA and collagen. In a preclinical study of streptozotozin-induced diabetic rats, a downregulation of TGF-β, collagen and α-SMA expression was observed following intraperitoneal UCSC injection [41].

Some studies published in 2022 used modifications in stem cell preparation or co-interventions aiming to enhance their therapeutic effect. He et al. evaluated the efficacy of ADSC co-modified with VEGF and Smad7 in a CNI rat model [42]. Erectile function in rats treated with co-modified ADSC was more distinctly recovered. Despite not being the subject of the present review due to their distinct origin from MSC, Zhuang et al. explored the effect of urine-derived stem cells (UDSC) administered in hyaluronic acid vesicles, observing an enhanced response in diabetic rats compared with only-UDSC administration. A similar enhanced response might be observed in modified preparations with hyaluronic acid with other cellular lines. This also represents an advantage regarding treatment-associated discomfort, as topical application is feasible.

### 3.2. Stem Cell Therapy in the Clinical Setting

Since 2010, a total number of 23 interventional studies have been registered in ClinicalTrials.gov aiming to evaluate the safety and efficacy of stem cell therapy in the treatment of ED, out of which 9 studies have already been completed. A summary of their main characteristics, measured outcomes, type of intervention and results is presented in Table 1.

The main sources of stem cells for therapeutic use in humans include bone marrow-derived stem cells (BMSC) and ADSC; nevertheless, MSCs derived from the umbilical cord (UCSC) and placenta (PSC) have also been used. The main cell delivery method carried out in the majority of published studies has been direct intracavernous injection. Most of the study protocols have been designed in a similar fashion. Firstly, the isolation of desired stem cells from bone marrow tissue or fat (adipocytes) of the patient in the case of autologous transplantation or from a healthy donor in allogenic transplantation is required. This process is usually followed by in vitro culture expansion of the isolated clonal cells. Finally, the obtained product can be used in two ways, either direct injection of stem cells into the target patient or, alternatively, the use of the stromal vascular factor (SVF), which is further isolated from the obtained stem cells.

In the majority of studies, mainly composed of pilot phase I/II clinical trials, the main measured outcome was the tolerability and safety profile of the overall effect on erectile function. In a general manner, no serious adverse effects were found derived from ICI of MSCs.

#### 3.2.1. Umbilical Cord-(UCSC) and Placenta-Derived (PSC) Stem Cells

The first human clinical trial published by Bahk et al. [43] in 2010 used, for the first time in an experimental setting, allogenic UCSC in diabetes mellitus-derived ED. ICI with 1.5 × 10^7^ cells was performed in seven men between the ages of 57 to 83. Most of the patients recovered matutine spontaneous erections in the period of a month, and these results were maintained over the period of 6 months of follow-up. Additionally, a decrease in the levels of blood glucose after two weeks of treatment initiation was also observed, which may explain a potential benefit of MSCs in the treatment of diabetes.

The safety profile of the treatment was not a measured outcome in this particular study; however, in a posterior phase I clinical trial published by Levy et al., this was thoroughly assessed [44]. In this study, eight patients suffering from complete PDEi non-responsive ED were followed up for 6 months. Treatment-derived adverse effects and hemodynamic and functional results after ICI of placenta-derived stem cells (PSC) were analyzed. No serious adverse events were noted and an improvement in mean penile arterial flow, which was maintained at 6 months after treatment initiation, was observed.

#### 3.2.2. Bone Marrow-Derived Stem Cells (BMSC)

Yiou et al. published a phase I/II clinical trial (INSTIN Study) in which they included a total number of 18 patients (12 in the first phase of the study) following radical prostatectomy [45,46]. Dose-escalating ICI of BMSC were employed. Authors concluded that a single autologous BMSC ICI was safe and effective in patients with vasculogenic ED; further improvements in perceived erectile function and sexual satisfaction were observed at the 6-month follow-up and were maintained over a 1-year period. The complete mean follow-up period of 62.1 months confirmed a complete absence of treatment-derived adverse effects; however, a slight worsening in erectile function was observed with respect to results obtained in the first year following treatment. The authors additionally concluded that further ICI may be required over time for maintenance of the therapeutic effect. In another study, Bieri et al. confirmed the safety profile and efficacy of BMSC using a medical device that was FDA approved for cellular delivery in a phase I clinical trial including PDEi non-responders [47]. In the field of diabetes mellitus-derived ED, Al Demour et al. carried out two consecutive clinical trials in diabetic patients exploring the use of autologous BMSC and allogenic Wharton’s Jelly-derived stem cells (WJSC) to treat ED. In the first trial, ICI of BMSC was found to be safe and effective, with a significant improvement in International Index of Erectile Function (IIEF-15) and Erection Hardness Score (EHS) questionnaires [48]. In the second, the use of two consecutive ICI of WJSC was explored for the first time in a total sample of 22 diabetic patients with ED. Positive results regarding tolerability, safety and efficacy measured via IIEF-15 and EHS questionnaires and duplex doppler penile ultrasound imaging (DPE) were observed at the 12-month follow-up [49].

**Table 1:** Experimental studies on the treatment of ED with MSC.

NCT	MI	Study Design	Design Adm./Dose Route	Comorbidity	Sample Size/Follow-Up (Months)	Latency Period until Improvement of ED	Duration Effect (Months)	SAE	Year of Publication
NR	UC-MSCs	Phase I/Pilot study. Single blinded	Single ICI/1.5 × 10^7^ Cells	Type II DM	10 (7)/9	1	11	No	2010 [43]
NR	BM-MNSCs	Case report	Single ICI/unquantified	COED	1/18	0.75	18	No	2013 [31]
NR	ADSC	Pilot study	Single ICI/1.5 × 10^7^ Cells	Type II DM	6/12	2	12	No	2015 [50]
02398370	PM-MSCs	Phase I-II/Open Label	Single ICI/unquantified	COED	8/6	1.5	6	No	2016 [44]
01089387	BM-MNSCs	Phase I	Single ICI/2 × 10^9^–2 × 10^7^ Cells	Post-RP	12/12	3	6	No	2016 [46]
		Phases II:	Single ICI/10 × 10^8^ Cells		18/62.1	3	6		2017 [45]
02240823	ADSC	Phase I/Open Label	Single ICI/8.4–37.2 × 10^6^ Cells	Post-RP	17/6	6	6	No	2016 [51]
					21/12	6	12		2018 [52]
02945462	BM-MSCs	Phase I/Open Label	Two ICI/30 × 10^6^ Cells	Type II DM	4/12–24	1	12	No	2018 [48]
NR	AD-MSCs/PLP	Open Label/Pilot study	Single ICI/47 × 10^6^ Cells	Type II DM, AHT, DL, Peyronie D	8/3	1	3	No	2019 [53]
NR	Trasnsendocardial hMSC	Retrosp. Cohort	20-20047 × 10^6^ Cells	Cardiomyopathy-ED	36/12	3	12	No	2019 [54]
NR	AD-MSCs/PLP	Phase I/Pilot Study. Open Label	Single ICI/47 × 10^6^ Cells	Type II DM, AHT, DL, Peyronie D	5/6	1	6	No	2020 [55]
NR	MSC-DE/LISWT	Prosp. Cohort	6 ICI/5 mL	Metabolic syndrome-ED	38/3		3	No	2020 [56]
03699943	ABMC	Phase I/Dose escalation	Two ICI 3–6 mL 10^8^ Cells	COED	40/12	3	6	No	2020 [47]
02945449	WJ-MSCs	Phase I/Open Label	Two ICI/20 × 10^6^ Cells	Type II DM	22/12	1	12	No	2021 [49]
023448499	BM-MSCs	Phase I/Open Label	ICI Cellgram ED/30 × 10^6^ Cells	DM/Post-RP	10	1	12	No	2021 [57]
NR	Oral mucosa-MSCs	Rand. Single-blinded	Single ICI/50–60 × 10^6^ Cells	DM-ED	20/6	3	6	No	2021 [58]
NR	SHED-CM	Open Label/Pilot study	3–8 ICI/unquantified	COED	38/8	ND	ND	No	2022 [59]
NR	ADSC	Prosp. case series	Single ICI/unquantified	COED	10/6	1	3	No	2022 [60]

UC-MSCs: umbilical cord-derived mesenchymal stem cells; BM-MNSCs: bone marrow-derived monuclear cells; ADSCs: adipose-derived stem cells; PM-MSCs: placental matrix-derived mesenchymal stem cells; WJ-MSCs: jelly-derived mesenchymal stem cells; AD-MSCs: adipose-derived mesenchymal stem cells; WJ-MSCs: Jelly-derived mesenchymal cells; AD-MSCs: adipose tissue-derived mesenchymal stem cells; BM-MSCs: bone marrow-derived mesenchymal stem cells; ABMC: Autologous Bone Marrow Concentrate; COED: Chronic Organic Erectile Dysfunction; ICI: Intracavernous Injection. SHED-CM: human exfoliated deciduous dental pulp stem cells; MSC-DE/LISWT: mesenchymal stem cell-derived exosomes/low-intensity shock wave therapy; Trasnsendocardial Hmsc: transendocardial human mesenchymal stem cells; NR: No register; ND: No data.

#### 3.2.3. Adipose-Derived Stem Cells (ADSC)

Another of the most studied sources of MSC for ED treatment, as we previously mentioned, is adipose tissue. ADSC are metabolically active cells with an important role in damaged tissue revascularization, apoptosis inhibition and immunomodulatory processes. They possess unique auto-regenerative and multipotential capabilities similar to BMSC and, unlike the latter, they can be obtained easily and in great quantity. Additionally, as previously exposed, they exert an important paracrine effect with the secretion of extracellular matrix factors, a great number of cytokines and other growth factors including VEGF and bFGF, all of them with angiogenic and antiapoptotic properties [22]. In a small study performed in 2015 by Garber et al., 1.5 × 10^7^ ADSC were injected into six diabetic patients [50]. ICI consisted of ADSC obtained after culture, not isolating the SVF. Four of the six patients recovered spontaneous morning erections in a one-month period, with the ability to perform sexual intercourse up to twelve months after ICI with the aid of PDEi treatment during the last month. These results are consistent with the published results from the Danish group led by Haahr, which demonstrated that ICI of autologous ADSC was a safe and effective treatment in patients with ED following radical prostatectomy [51]. No adverse events were observed at the 12-month follow-up. IIEF-15 scoring results improved significantly at 6 months following treatment, and the improvement persisted at 12 months [52]. It is important to note that this improvement was observed in patients with a normal preoperative erectile function and urinary continence at the time of inclusion. An ongoing phase III study by this same working group has been authorized and is currently under production in a much larger sample of post-prostatectomy subjects.

On the other hand, Protogenau et al., in an effort to explore and replicate the potential benefit of substances present in SVF, have come up with a technique of combined administration with ADSC. A combination of cultured ADSC suspended in platelet-rich plasma lysate (PLP) was administered. Obtained results were promising; treatment improved erectile function after one-, three- and six-month follow-up without presenting negative adverse effects [53,55].

Similar results to the recent clinical study with adipose-derived stem cells were published by Fode et al. [60] A prospective case series of 10 men was conducted to investigate the feasibility and safety of a new minimally invasive same-day method of autologous adipose-derived stem cell (ADSC) transplantation using the myStem^®^ X2 kit.

#### 3.2.4. Other Stem Cell Sources

Most of the published studies have used stem cells derived from bone narrow and adipose tissue, but other types of stem cells from various strains have been tested.

A retrospective study by Ory et al. analyzed the effect of stem cells with the use of transendocardial human mesenchymal stem cells (hMSCs) injection as a treatment in cardiomyopathy patients who experienced erectile dysfunction [54]. The hMSCs were autologous and allogeneic with varying doses of 20, 100 and 200 million cells. This post hoc analysis was the first to investigate the effect of SCT on erectile function using randomized, placebo-controlled data.

The study by Zasieda et al. reported the use of mesenchymal stem cell-derived exosomes (MSC-DE) injected intravenously once per week for six weeks, a combined MSC-DE injection with low-intensity shock wave therapy (LISWT) twice per week, 3000 strikes and 3 Hz frequency. The IIEF score significantly increased compared to baseline and improved peak systolic velocity and reduced end diastolic penile velocity significantly posttherapy [56].

In 2021, Mirzaei et al. used an intracavernous injection of stem cells isolated from the oral mucosa in a dose of 50–60 million cells [58]. The results of this randomized clinical trial with a control group concluded that intracavernous stem cell injection improved sexual function and the PSV and RI indices of penile arteries in diabetic patients, with no side effects observed.

Finally, Koga et al. [59], aiming to investigate the recovery of sexual function through cellular regeneration of damaged vascular tissue in the corpus cavernosum, tested direct injection of stem cells derived from the exfoliated deciduous dental pulp (SHED-CM). The results after the treatment of 38 ED patients without prior treatment with PDE5I or testosterone replacement therapy showed a statistically significant improvement in IIEF-5 scores after three courses of SHED-CM treatment. However, no pathological assessment was performed to accurately judge the effects of SHED-CM on vascular endothelial cells and its effect over follow-up. The safety of SHED-CM treatment, as well as its potential to repair vascular damage to the corpora cavernosa, represents a potential future treatment for patients with erectile dysfunction.

### 3.3. Ethical Concerns and Limitations

ED is a urological disorder with an important psychological burden for those suffering from this condition. Well-being, self-esteem and social relationships are profoundly affected, as sexual function is an intricate part of good individual health. Despite basic principles in the treatment of ED that can be applied to all men, the restoration of sexual function in an individual and their sexual partners must be considered in an individual manner. Therefore, we must provide these patients with measured evidence and realistic expectations derived from treatment. It is also important to take into consideration, in the specific field of issues in sexuality, the strength of the placebo effect. It is clear that patients in the placebo arm of studies on ED treatment usually present statistically significant improvement in their self-perceived erectile function [61]. In single-arm published studies, this effect should be heavily considered as a possible source of bias.

Lastly, the negative impact of these kind of therapies in the so-called predatory health-related tourism needs to be highlighted. Stem cell therapy for ED should only be considered in an experimental setting. Despite multiple warnings from the European Medicines Agency (EMA) and the Food and Drug Administration (FDA), this type of tourism grows exponentially over time [62]. As we previously mentioned, treatment results in the field of ED are rather subjective; thus, these practices are easier to perform on a tremendously influential and desperate patient population leading to unevidenced, lucrative and potentially dangerous interventions [63].

## 4. Discussion

In a general manner, interventional studies on the treatment of ED with stem cell therapy have shown promising results with observed improvements in erectile function. These improvements may be measured as hemodynamic parameters (mean penile arterial flow) or via sexual function questionnaires (IIEF-15 or EHS). Even though most of the studies support the safety profile and treatment efficacy of ICI of MSC, the available studies are difficult to standardize. Evidence to this day mainly consists of small, open-label, single-arm trials in which the MSC of different sources has been employed, with significantly distinct study protocols. Therefore, it is difficult to establish conclusive remarks without the existence of more conclusive and homogenized data on the matter. In response, most of the studies conclude with the idea that further research on stem cell therapy for ED is required in order to establish as a contrasted treatment modality for these patients in everyday practice.

Stem cell therapy is an experimental treatment modality. Due to its experimental nature, there is a lack of use and manufacturing standardization, leading to possible differences between stem cell processing laboratories. Diversity may lead to inconsistent and variable responses, as well as poor replicability in the experimental setting. Efforts should be made to optimize of MSC manufacturing and processing, establish quality standards and promote legislative measures for a better outcome and homogeneity. In an effort to provide a guide for better and homogeneous manufacturing of therapeutical cellular products focusing on MSCs, a recent review has been published [64]. Authors propose four critical steps in MSC manufacturing for their application as therapeutical products. These four steps include: adequate donor selection, harvesting–isolation–expansion of MSCs, storage–distribution related issues and cell handling. Regarding donor selection, both the tissue of origin and the donor person should be considered. The use of possible of autologous MSCs should be considered; however, if donor cells are used, they should undergo certain quality check controls prior to their use. Active or latent infection in the donor cells should be ruled out [65]. Additionally, a particular cell product could possess optimal characteristics for one indication but insufficient for another. Different approaches have been made in order to match different cellular lines with potential indications according to some of their properties, such as proliferative and differentiation capacity or secretory function in relation to specific conditions and donor–receptor characteristics. MSCs’ isolation and expansion standards include adequate source selection, the choice for the best and less morbid collection technique and optimal culture conditions for expansion following well-defined standard operating procedures (SOPs) that need consecutively approved validations. On the other hand, the use of cell banks guarantees available tissue without the need for new biopsies, and cryopreservation improves the quality of storage and distribution. Adequate cell handling involves selecting the optimal administration route according to the specific type of cellular line used and establishing certain regulations with the aid of Hospital Pharmacy Department or Cell Therapy Area if available.

Another important aspect regarding the treatment of ED is the increasing economic cost it represents for Public Health Services, which exponentially grows as more treatment modalities are used due to previous treatment failure [66]. Thus, it is important to search for new treatment strategies that not only symptomatically treat but also “cure” a pathological condition. In this sense, further research is required to consider MSC a standard treatment for ED, as preliminary data reflect this curative nature associated with their use. If feasible, integration into Public Health Services as an option of treatment will reflect an enormous economic impact, as well as assuring well-being and added safety for the general population, avoiding predatory and potentially dangerous practices once it is regularized and practiced in licensed centers.

Large-scale human multicenter studies evaluating the efficacy of stem cell therapy in ED, ensuring adequate statistical power, are required to establish definitive conclusions. There is a large number of basic research published on the matter, in vitro and in vivo animal models; however, human research is lacking. Due to the potential bias derived from the placebo effect in research involving self-perceived improvements in sexual function, larger and comparative studies are needed. Intervention groups should be compared not only against placebo but should also include treatment modalities already established in clinical guidelines to elucidate the real therapeutical effect. Further comparisons between different stem cell therapy modalities, such as cellular and cell-free (SVF) are also required.

## 5. Conclusions

Stem cell therapy for the treatment of ED represents a promising potential therapy. However, conclusive high-level evidence is lacking, and these types of interventions should only be considered in an experimental setting. Results from basic research and animal models are impressive, but more human research is required in order for it to be included as an option for the treatment of ED.

## Data Availability

Not applicable.
